# Friend Spleen Focus-Forming Virus Activates the Tyrosine Kinase sf-Stk and the Transcription Factor PU.1 to Cause a Multi-Stage Erythroleukemia in Mice

**DOI:** 10.3390/v2102235

**Published:** 2010-10-11

**Authors:** Joan Cmarik, Sandra Ruscetti

**Affiliations:** Laboratory of Cancer Prevention, National Cancer Institute, Frederick, MD 21702, USA; E-Mail: cmarikj@mail.nih.gov

**Keywords:** friend spleen focus-forming virus, leukemia, tyrosine kinases, sf-Stk, Epo receptor, PU.1, signal transduction pathways

## Abstract

Hematological malignancies in humans typically involve two types of genetic changes: those that promote hematopoietic cell proliferation and survival (often the result of activation of tyrosine kinases) and those that impair hematopoietic cell differentiation (often the result of changes in transcription factors). The multi-stage erythroleukemia induced in mice by Friend spleen focus-forming virus (SFFV) is an excellent animal model for studying the molecular basis for both of these changes. Significant progress has been made in understanding the molecular basis for the multi-stage erythroleukemia induced by Friend SFFV. In the first stage of leukemia, the envelope protein encoded by SFFV interacts with and activates the erythropoietin (Epo) receptor and the receptor tyrosine kinase sf-Stk in erythroid cells, causing their Epo-independent proliferation, differentiation and survival. In the second stage, SFFV integration into the S*fpi1* locus activates the myeloid transcription factor PU.1, blocking erythroid cell differentiation, and in conjunction with the loss of p53 tumor suppressor activity, results in the outgrowth of malignant cells. In this review, we discuss the current level of understanding of how SFFV alters the growth and differentiation of erythroid cells and results in the development of erythroleukemia. Our knowledge of how SFFV causes erythroleukemia in mice may give us clues as to how the highly related human retrovirus XMRV causes malignancies in humans.

## 1. Introduction

Fifty-three years ago, Charlotte Friend described an acute erythroleukemia induced in adult Swiss mice by a viral preparation that subsequently became known as Friend virus [1]. It was later shown that this virus preparation contained two retroviruses: (1) a defective spleen focus-forming virus (SFFV), which is responsible for the acute erythroleukemia that occurs in adult mice, and (2) a replication competent Friend murine leukemia virus (F-MuLV), which is not pathogenic by itself in adult mice, but serves as a helper to allow the defective SFFV to infect and integrate into cells. SFFV was derived from recombination of Friend MuLV with endogenous polytropic envelope gene sequences in the mouse, generating in the process a retrovirus that contains deletions in all of its structural genes and a unique envelope gene [2,3].

**Figure 1:**
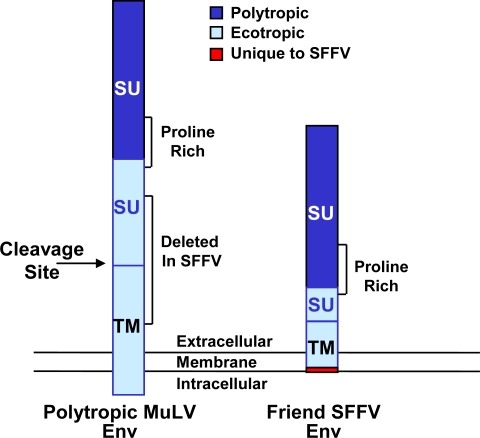
Comparison of the SFFV envelope protein with that of a polytropic MuLV. The unique envelope glycoprotein encoded by SFFV is the sole determinant of its pathogenicity. The envelope precursor of a typical polytropic MuLV is processed to SU and TM proteins. In contrast, the SFFV envelope glycoprotein (Env), due to a large deletion that eliminates the cleavage site between SU and TM, as well as a single base pair insertion in TM, contains fused SU and TM sequences, has a unique transmembrane region and lacks a cytoplasmic tail.

SFFV, unlike most acutely transforming retroviruses, does not carry an oncogene but derives its oncogenic potential from the product of its unique envelope gene, gp55 [2,3]. Although the SFFV envelope gene is closely related to that of the polytropic Friend mink cell focus-inducing MuLV, it contains several changes that are responsible for its unique biological effects (Figure 1) [4–6]. First of all, it contains a large deletion in the open reading frame of the gene that eliminates a proteolytic cleavage site where the envelope protein precursor of a typical MuLV is cleaved into surface unit (SU) and transmembrane (TM) proteins. Thus, SFFV gp55 is a fusion protein containing the N-terminal domain of SU and the C-terminal domain of TM. Secondly, a single base pair insertion in the TM-encoding region of the SFFV *env* gene results in a change in reading frame. This causes the loss of the cytoplasmic tail that is present in a typical MuLV envelope protein and creates a unique C-terminus of 5–6 amino acids. Each of these changes in the SFFV *env* gene are required for pathogenicity [7–10]. Unlike a typical MuLV envelope protein, SFFV gp55 does not function as a structural protein in the virion. It is inefficiently transported to the plasma membrane, with only about 5% of the protein leaving the endoplasmic reticulum as a disulfide-bonded dimer, that is further processed through the Golgi to a dimer of 65 kD subunits, which appears on the cell surface [11–14]. The cell surface form of the SFFV envelope protein appears to mediate the biological effects of the virus [15–17].

## 2. Biological Effects of SFFV

**Figure 2:**
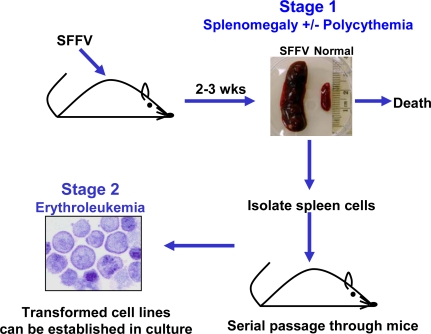
SFFV induces a multistage erythroleukemia in mice. Stage 1 is the preleukemic stage and is characterized by erythroid hyperplasia and in some cases polycythemia. Stage 2, which can best be detected after serial transplantation of Stage 1 cells through syngeneic mice, represents the leukemic stage of the disease. SFFV-infected erythroid cells from Stage 2 can be established *in vitro* as transformed cell lines.

The biological effects of SFFV are specific for the erythroid lineage and can be divided into two distinct stages (Figure 2). The first is a preleukemic stage that is the direct result of expression of the SFFV envelope protein (Env) in erythroid precursor cells. This causes their uncontrolled proliferation leading to rapid erythroid hyperplasia characterized by grossly enlarged spleens and livers. With some strains of SFFV, erythropoietin (Epo)-independent differentiation of erythroid cells also occurs, resulting in polycythemia. Death, which occurs within three weeks after virus infection, is thought to be the result of splenic rupture. The second stage of SFFV-induced disease represents the transformation phase and can be detected only late after virus infection or after serial passage of the leukemic splenocytes through syngeneic mice. These SFFV-transformed erythroid cells form macroscropic colonies in methylcellulose [18] and can be grown as immortal erythroleukemia cell lines in culture. Since SFFV is a replication defective virus, studies have routinely been carried out in the presence of helper virus. However, the helper virus does not contribute directly to either the erythroid hyperplasia or the transformation of erythroid cells induced by the virus complex, since both effects can be reproduced using a helper-free preparation of SFFV [19–22].

### 2.1. Induction of Erythroid Hyperplasia by SFFV

**Figure 3:**
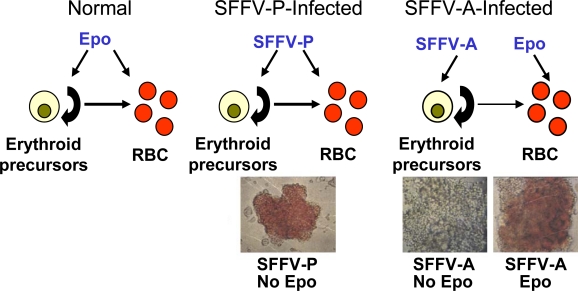
Epo-independent erythroid proliferation and differentiation induced by SFFV. Normal erythroid cells require Epo for proliferation and differentiation into red blood cells (RBC). However, erythroid cells infected with SFFV-P or SFFV-A *in vitro* can proliferate in the absence of Epo, as shown by the development of Epo-independent erythroid colonies. Those infected with SFFV-P, but not SFFV-A, are red, indicating that differentiation also proceeds in the absence of Epo.

The hallmark of the first stage of SFFV-induced leukemia is the induction of Epo-independence. While normal erythroid cells require Epo for their proliferation and differentiation, those infected with SFFV can proliferate and, depending upon the strain of SFFV, differentiate in the absence of Epo. This can be demonstrated *in vitro* by infecting primary erythroid progenitors with SFFV, which form colonies in the absence of Epo (Figure 3). Erythroid cells infected *in vitro* with SFFV-P, a strain of the virus which causes erythroblastosis as well as polycythemia in mice, can both proliferate and differentiate in the absence of Epo [23,24], as indicated by the induction of Epo-independent hemoglobinized colonies. In contrast, erythroid cells infected *in vitro* with SFFV-A, a strain that causes erythroblastosis without polycythemia in mice, can proliferate in the absence of Epo but still require Epo for their differentiation. [25,26]. SFFV can also render Epo-dependent hematopoietic cell lines factor-independent [27–29]. The Epo-dependent erythroleukemia cell line HCD-57 can grow in the absence of Epo after infection with either SFFV-P or SFFV-A [29], but only SFFV-P can confer factor-independence to BaF3-EpoR cells, an IL-3 dependent hematopoietic cell line that has been engineered to express the Epo receptor (EpoR) [27,28,30]. The different biological phenotypes conferred by SFFV-P and SFFV-A have been attributed to a 113 bp region encompassing the membrane spanning domain of the viral envelope protein [31]. This transmembrane region contains several amino acid differences between the two viruses, including methionine at position 390 and two extra leucine residues in the envelope protein of SFFV-P [32], both of which are required for the development of polycythemia in mice and to render BaF3-EpoR cells Epo-independent [33,34].

Not all strains of mice are susceptible to erythroleukemia induction by SFFV. Some strains of mice carry genes that prevent the helper virus from infecting or integrating into the target cell, while others express genes that lead to the development of an effective immune response against the virus or virus-infected cells [35]. In addition, certain resistant strains of mice have genetic defects that alter the number of erythroid target cells for the virus. The only host gene that controls the susceptibility of erythroid target cells to SFFV is the *Fv2* gene [36]. The *Fv2* gene encodes the receptor tyrosine kinase Stk, and mice must encode a unique form of this kinase to be susceptible to SFFV-induced erythroleukemia (see Section 3.3.) [37].

### 2.2. Transformation of Erythroid Cells by SFFV

The majority of the SFFV-infected erythroid cells proliferating in the first stage of SFFV-induced disease have a finite self-renewal capacity. However, SFFV is capable of transforming erythroid cells, although the transformed cells are detectable only in the late stages of disease. Since most SFFV infected mice die before these transformed cells gain a growth advantage, the virus infected spleen cells proliferating during the erythroblastosis phase of the disease are typically serially transplanted to syngeneic mice to enrich for the transformed cells (see Figure 1). Unlike erythroid cells present in the early stages of SFFV-induced disease, SFFV-transformed cells, called murine erythroleukemia (MEL) cells, are monoclonal and are thought to represent the progeny of a single cell that has undergone secondary genetic events. MEL cells are capable of growing as subcutaneous tumors in mice and as immortal erythroleukemia cell lines *in vitro* [38–40]. SFFV-transformed MEL cells, unlike those proliferating in the first stage of SFFV-induced disease, are blocked in differentiation, although they can be induced to differentiate into red blood cells with certain chemicals, but not with Epo [41].

The mechanisms by which SFFV and its unique envelope protein cause multi-stage erythroleukemia in adult mice have been the subject of numerous investigations. Below, we will discuss our current level of understanding of how expression of SFFV in erythroid cells leads to deregulation of signal transduction pathways to cause erythroid cell hyperplasia, polycythemia and transformation.

## 3. Molecular Basis for the Epo-independent Erythroid Hyperplasia induced by SFFV

Two key characteristics of the first stage of SFFV-induced disease are the higher than normal rate of proliferation of erythroid cells and the lack of dependence on Epo for this hyperplasia to occur. Studies have shown that Epo-independent proliferation, differentiation and survival of SFFV-infected erythroid cells directly results from constitutive activation of signal transduction pathways that is mediated by the interaction of the SFFV envelope glycoprotein with two molecules: the EpoR and a short form of the receptor tyrosine kinase Stk.

### 3.1. Constitutive Activation of Signal Transduction Pathways by SFFV

Because infection of erythroid cells with SFFV mimics some of the effects of Epo on those cells, numerous investigations logically focused on determining whether the unique envelope glycoprotein of SFFV, which is the sole determinant of its pathogenicity, was able to activate signaling molecules regulated by the interaction of Epo with its dimeric cell surface receptor. Through either phosphorylation of the EpoR or of adapter molecules, binding of Epo to the EpoR activates several distinct pathways, including the Ras/Raf/MAP kinase, the Jak-Stat, and the PI 3-kinase pathways [42]. As was predicted, comparisons of Epo-dependent erythroid cell lines and their SFFV-infected counterparts revealed that SFFV activates these same signal transduction pathways. In contrast to Epo-stimulated erythroid cells, however, the EpoR is not constitutively phosphorylated in SFFV-infected cells [43], indicating that SFFV is activating signals primarily through a redundant EpoR pathway involving the activation of adapter molecules, such as members of the IRS family of adapters [44,45]. Table 1 summarizes the findings described below regarding activation of signaling molecules of the Epo/EpoR pathway by both SFFV-P, which causes Epo-independent proliferation and differentiation, and SFFV-A, which causes only Epo-independent proliferation.

Studies of the Ras/Raf/MAP kinase pathway show that upstream events (including Shc phophorylation and Shc-Grb2 complex formation), Ras activation, and activation of downstream components (including Raf-1, MEK, and the MAP kinases ERK1/2 and JNK) occur constitutively in both SFFV-P and SFFV-A-infected cells [46–48]. While necessary for the proliferation of erythroid cells in response to Epo, Raf and Ras are not required for the proliferation of SFFV-infected erythroid cells, although proliferation was reduced in the absence of Raf and Ras [46,47]. In contrast, the Epo-independent proliferation of SFFV-infected cells absolutely requires activation of ERK and JNK [48,49], suggesting that SFFV also activates a Ras/Raf-independent MAP kinase pathway. Phosphorylation and translocation to the nucleus of Stats 1, 3 and 5 occurs in SFFV-P-infected erythroid cells in the absence of Epo [50,51]; constitutive Stat activation occurs without tyrosine phosphorylation of the EpoR and may not require Jak2 activation [43]. In contrast to SFFV-P, SFFV-A infection of erythroid cells does not result in the activation of Stats 1 and 5, although Stat3 is constitutively activated [52,53]. Thus, activation of Stats 1 and 5 may be necessary for the Epo-independent induction of erythroid cell differentiation, which does not occur after infection of erythroid cells with SFFV-A. Stat5 increases transcription of the anti-apoptotic protein Bcl-X_L_ in erythroid cells in response to Epo [54], and Bcl-X_L_ may also protect SFFV-infected cells from apoptosis after Epo withdrawal. Unlike normal erythroid cells, SFFV-P-infected erythroid cells express high levels of this anti-apoptotic protein even after Epo withdrawal [55].

Both SFFV-P and SFFV-A also activate the PI 3-kinase pathway. Constitutive PI 3-kinase activation in SFFV-infected cells results not from its association with the EpoR, which is not tyrosine phosphorylated in SFFV-infected cells, but from the association of its p85 regulatory subunit with the constitutively phosphorylated adaptor molecules IRS-2, Gab1 and Gab2 [43]. PI 3-kinase activity is required for SFFV-induced erythroid cell proliferation: pharmacological inhibition of PI 3-kinase activity blocks the Epo-independent proliferation of SFFV-infected cells [43,49], and SFFV-induced splenomegaly is attenuated in p85α-deficient mice [56]. The serine/threonine protein kinases Akt and PKC, downstream effectors of PI 3-kinase, are constitutively phosphorylated in SFFV-infected cells [43,47]. MEK is one of the targets activated downstream of PKC; thus, PKC activation could explain the Ras/Raf-independent activation of ERK in SFFV-infected cells. In contrast to Ras and Raf, PKC is absolutely required for SFFV-induced ERK activation and cell proliferation in the absence of Epo [47]. Activation of both PKC and Akt may promote survival of SFFV-infected erythroid cells in the absence of Epo. In other systems, PKC has been shown to increase levels of the anti-apoptotic protein Bcl-X_L_ [57], and Akt phosphorylates BAD [58], thereby inactivating this pro-apoptotic Bcl-2 family member.

Thus, analogous to the effects of Epo on erythroid cells, SFFV activates multiple signal transduction pathways that stimulate their growth and differentiation. Activation of MAP kinases, PI 3-kinase, PKC and Stat3 are required for Epo-independent proliferation. In contrast, Stats 1 and 5 appear to be involved in the induction of SFFV-induced Epo-independent differentiation. Protection of SFFV-infected erythroblasts against apoptosis may be conferred through the activation of Stat5, PKC and Akt. By activating all of these signals, SFFV-P-infected erythroblasts circumvent the normal regulation of proliferation, differentiation, and survival, leading to the observed rapid erythroblastosis and polycythemia in affected animals.

### 3.2. Interaction of SFFV Env With the EpoR

SFFV Env has no homology to Epo or its receptor, no intrinsic kinase activity, no DNA-binding motif, and lacks a cytoplasmic tail to dock signal transducing molecules. Thus, the leading hypothesis for its mode of action on erythroid cells was that it interacted with a component(s) of the Epo signal transduction pathway at the cell surface that would mediate proliferation and differentiation in the absence of Epo. The main candidate for such an interaction was the EpoR. Strong support for this hypothesis came from studies demonstrating that the IL-3-dependent cell line BaF3 could be converted to factor-independence by SFFV-P infection if and only if a construct driving exogenous expression of the EpoR was introduced [28]. Cross-linking studies with iodinated Epo revealed that SFFV Env was in close proximity to the EpoR at the cell surface [15,59], establishing that the viral envelope protein is in a position where it could alter Epo signal transduction pathways. Co-immunoprecipitation experiments demonstrated that an extracytoplasmic region of the EpoR specifically interacts with SFFV Env, and studies using chimeric receptors showed that the transmembrane region of the EpoR is critical for activation by SFFV Env and for subsequent biological events to occur [60]. Further studies showed that serine 238 in the transmembrane region of the EpoR interacts with methionine 390 in the envelope glycoprotein of SFFV-P, and it is thought that these specific transmembrane interactions induce receptor oligomerization and a conformational change in the EpoR like that induced by Epo [33,61]. Although the murine and human EpoRs are closely related, the envelope protein of SFFV-P fails to activate the human EpoR, which contains leucine instead of serine at position 238 [33]. Unlike SFFV-P, the envelope glycoprotein of SFFV-A cannot interact with and activate the EpoR, and this is attributed to the presence of isoleucine at position 390 in SFFV-A Env [33].

Although the envelope glycoprotein of SFFV-P can interact with and activate the EpoR, this interaction does not appear to be necessary for the development of Epo-independent erythroid cell hyperplasia. SFFV-A, which encodes an envelope protein that cannot activate the EpoR, still causes Epo-independent erythroid cell hyperplasia in mice, although the animals do not develop polycythemia [25,26]. Furthermore, mice in which the mouse EpoR has been replaced with the human EpoR, which cannot be activated by SFFV Env [33], still develop SFFV-P-induced erythroblastosis but not polycythemia [52]. Thus, interaction of SFFV-P Env with the EpoR appears to be responsible for the development of Epo-independent erythroid differentiation and polycythemia and not required for the development of SFFV-induced erythroid cell hyperplasia.

### 3.3. Interaction of SFFV Env with the Receptor Tyrosine Kinase sf-Stk

Since the envelope protein of SFFV lacks any kinase activity, the Epo-independent tyrosine phosphorylation of various signal transduction molecules in SFFV-infected cells must involve activation of a cellular tyrosine kinase. Although the tyrosine kinase Jak2 plays an important role in tyrosine phosphorylation of the EpoR in response to Epo in normal erythroid cells [62], Epo-independent EpoR phosphorylation is not observed in SFFV-infected cells, and it is unclear whether Jak2 is constitutively activated in these cells [43,52]. Thus, some other kinase must be responsible for the multiple signal transducing molecules that are tyrosine phosphorylated in SFFV-infected erythroid cells in the absence of Epo. Constitutive activation of several other tyrosine kinases that are known to be activated by Epo, including Fes and Tec, was not detected in SFFV-infected cells [43]. Although the tyrosine kinase Lyn was shown to be constitutively activated in SFFV-infected erythroid cells, it was not required for the induction of Epo-independent erythroid colonies by SFFV or for the development of SFFV-induced erythroleukemia [63].

**Figure 4:**
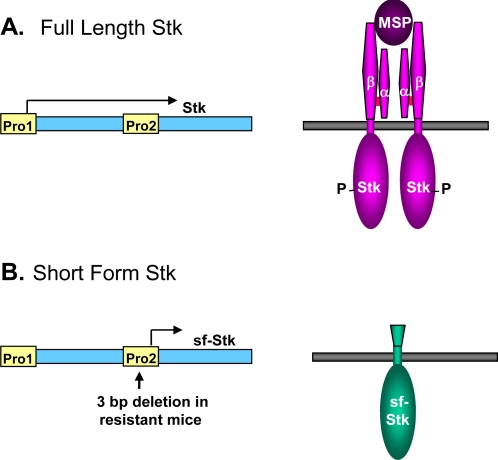
Comparison of full length and short form Stk. The *Mst1r* gene generates a full length transcript which encodes Stk, a tyrosine kinase that serves as the cell surface receptor for macrophage stimulating protein (MSP). The *Mst1r* gene also uses an internal promoter (Pro2) to encode a short form of Stk (sf-Stk), which lacks the MSP binding domain and whose function is unknown. Strains of mice resistant to SFFV-induced erythroleukemia do not encode sf-Stk due to a 3 bp deletion in the internal promoter. Although sf-Stk lacks a signal sequence, it is shown in the membrane for comparison.

Eliminating a role for kinases known to be activated by Epo pointed to the possibility that some kinase typically not associated with the Epo/EpoR pathway might be involved. This indeed turned out to be the case; the responsible kinase is a short form Stk (sf-Stk). The identification of the kinase was the outcome of studies to understand the host gene *Fv2*, which confers susceptibility to SFFV-induced erythroleukemia at the level of the erythroid target cell. The *Fv2* gene was shown to encode the Met-related receptor tyrosine kinase Stk [37]. Stk is the receptor for macrophage stimulating protein, which regulates macrophage motility [64]. The Stk gene, *Mst1r*, is expressed as a full length message, encompassing all 19 exons of the gene [65], and, in some cases, also as a shorter message transcribed from an internal promoter in intron 10 of *Mst1r*. The short message only contains exons 11–19, thus the encoded sf-Stk protein contains the transmembrane and tyrosine kinase domains of Stk but lacks most of its extracellular domain, including the ligand binding domain (see Figure 4) [66]. Although all strains of mice express the full length form of the kinase, only those strains susceptible to SFFV-induced erythroleukemia express sf-Stk [37]. Resistant strains of mice fail to express the short form due to a 3 bp deletion that includes the 5’-most nucleotide of the short transcript and that disrupts a GATA-1 transcription factor binding site in the internal promoter from which this transcript is expressed. Wild mice of the genus *Mus spretus* are also resistant to SFFV-induced disease, and although the sequence of their Mst1r gene includes the 3 bp deletion found in resistant laboratory strains, it also contains a point mutation in a Myb transcription factor binding site within the intron 10 promoter [37]. Consistent with the erythroid specificity of SFFV-induced disease, erythroid cells are one of the few types of cells that abundantly express sf-Stk [66]. The physiological role of sf-Stk is unknown. Exogenous expression of sf-Stk in *Fv2* resistant mice is sufficient to make them susceptible to SFFV disease [37]. By virtue of its requirement to activate SFFV-induced Epo-independent signaling in erythroid cells and erythroleukemia in mice, sf-Stk became a likely candidate to mediate the activation of signal transduction pathways by SFFV, and subsequent studies focused on confirming this hypothesis.

Sf-Stk is phosphorylated in leukemic splenocytes from SFFV-infected mice, but not those from uninfected mice [67], suggesting that SFFV activates sf-Stk. A direct covalent interaction between SFFV Env and sf-Stk was demonstrated when sf-Stk and SFFV Env were co-expressed in the BaF3-EpoR cell line [68]. In contrast, a weakly pathogenic mutant of SFFV, BB6, fails to interact with and activate sf-Stk. BB6 lacks two of the four cysteines in the ecotropic extracellular domain of SFFV-P Env, suggesting that these cysteines in SFFV-P Env may be responsible for disulfide bond formation with those present in the extracellular domain of sf-Stk [68]. Recent studies confirm that the interaction of SFFV Env and sf-Stk requires disulfide bond formation between at least two of the cysteines of the ecotropic domain of SFFV Env and cysteines of the extracellular domain of sf-Stk [69]. Interaction of SFFV Env and sf-Stk prolongs the half-life of sf-Stk [67] and results in sf-Stk phosphorylation and its association with multiple tyrosine-phosphorylated proteins [43]. SFFV Env does not interact with full-length Stk [43], possibly due to steric hindrance.

The interaction between SFFV Env and sf-Stk is critical for the induction of Epo-independence by the virus. Erythroid progenitors from *Fv2* resistant (*i.e.* sf-Stk null) mice, which do not form erythroid colonies *in vitro* in the absence of Epo, are able to form Epo-independent colonies following SFFV infection if they are first engineered to express sf-Stk [49], or if they are infected with a bicistronic retroviral vector allowing coexpression of SFFV Env and sf-Stk [67]. Sf-Stk alone does not induce Epo-independent erythroid colony formation. A constitutively activated mutant of sf-Stk induces Epo-independent erythroid colony formation in the absence of SFFV Env [67], further supporting the hypothesis that interaction with SFFV Env activates sf-Stk kinase activity.

Mutational analyses of sf-Stk have revealed domains required for the induction of Epo-independence. First and foremost, the tyrosine kinase domain is required; a kinase-dead mutant of sf-Stk could not induce Epo-independent colonies in conjunction with SFFV [49,67]. In addition, the most C-terminal tyrosine (amino acid 1337), which is one of two tyrosines comprising a multifunctional docking site, is essential [49,67]. The binding of the adapter molecule Grb2 to this tyrosine is important for the induction of Epo-independent erythroid colonies by SFFV-P [49], and haploid insufficiency of Grb2 decreases the susceptibility of mice to SFFV-P [70]. After binding to SFFV Env-activated sf-Stk, Grb2 recruits Gab2, another adapter found to be tyrosine phosphorylated in SFFV-infected cells [70]. Phosphorylated Gab2 can recruit SHP-2 and the p85 regulatory subunit of PI 3-kinase, molecules important for the SFFV-induced activation of the Ras/MAP kinase and PI 3-kinase pathways, respectively. This is supported by recent data indicating that PI 3-kinase p85α can be co-immunoprecipitated with sf-Stk. This co-immunoprecipitation requires sf-Stk kinase activity and tyrosine 436 in the multifunctional docking site of the kinase, suggesting that the interaction occurs indirectly via phosphorylated adaptor proteins such as Gab2 [56]. A recent study described a novel binding site by which Gab2 can also recruit Stat3 [71]. Interestingly, sf-Stk kinase activity is not required for the phosphorylation of Gab2 or Stat3 following their recruitment to the receptor [71]; these observations suggest that sf-Stk may be acting as a scaffold for these adapters rather than causing their phosphorylation. Although Gab2 null mice still develop erythroleukemia after infection with SFFV-P, progression of the disease is much slower, with significantly less splenomegaly observed at two weeks after infection [70], similar to that observed in SFFV-P-infected mice deficient in the p85α subunit of PI 3-kinase [56]. Thus, Grb2, Gab2 and Stat3 are all important mediators of signals coming from SFFV Env-activated sf-Stk, which seems to serve dual roles as both kinase and scaffold.

It is of interest to consider when, where, and how SFFV Env and sf-Stk interact within an infected cell. SFFV Env forms dimers in the endoplasmic reticulum that migrate through the Golgi to the cell surface [11,13]. Thus, each SFFV Env dimer could bind two molecules of sf-Stk. Although this might be a mechanism for bringing two molecules of sf-Stk close enough together for transphosphorylation to occur, recent data demonstrate that oligomerization of sf-Stk occurs independently of SFFV Env [69]. However, phosphorylation of sf-Stk is dependent upon SFFV Env [68,69]. Sf-Stk lacks an N-terminal signal sequence, and its interaction with SFFV Env is responsible for redirecting it from the cytosol to the cell membrane [69]. SFFV Env could stimulate the phosphorylation of sf-Stk by causing a conformational change in sf-Stk, possibly enhancing autophosphorylation, and/or by bringing sf-Stk to a region of the cell membrane where it can interact with another kinase.

Activation of sf-Stk, in contrast to activation of the EpoR, is absolutely required for the development of SFFV-induced erythroblastosis, indicating that signals initiated by this virus-activated kinase are essential. Not only can SFFV Env activate sf-Stk in erythroid cells, it can activate the kinase in rodent fibroblasts that have been engineered to express sf-Stk, and this activation can be measured by observing transformation of the fibroblasts. Since fibroblasts do not express the EpoR, these sf-Stk-expressing cells have been beneficial in studying signals resulting from SFFV Env-activated sf-Stk in the absence of EpoR signaling [72]. The envelope proteins of both SFFV-P and SFFV-A can transform sf-Stk-expressing rodent fibroblasts [72], indicating that both of these viral proteins can interact with and activate sf-Stk. This result is consistent with the ability of both SFFV-P and SFFV-A to cause erythroleukemia in mice. As previously observed in SFFV-infected erythroid cells, interaction of SFFV Env and sf-Stk in fibroblasts results in the activation of components of the MAPK and PI 3-kinase pathways, and these pathways are required for anchorage-dependent and -independent proliferation of the transformed cells [73]. Stat3 is also activated in the transformed rodent fibroblasts [73], consistent with studies indicating that Stat3 is activated downstream of sf-Stk in erythroid cells [71]. The p38 MAP kinase stress pathway, which is suppressed in SFFV-infected erythroid cells [55], is also suppressed in fibroblasts co-expressing SFFV Env and sf-Stk [73]. The only signal transducing molecules activated in SFFV-infected erythroid cells that are not activated in fibroblasts co-expressing SFFV Env and sf-Stk are Stats 1 and 5 [73], suggesting that activation of these Stats may require the involvement of the EpoR.

### 3.4. Different Roles of EpoR Signaling and sf-Stk in SFFV-induced Disease

**Figure 5:**
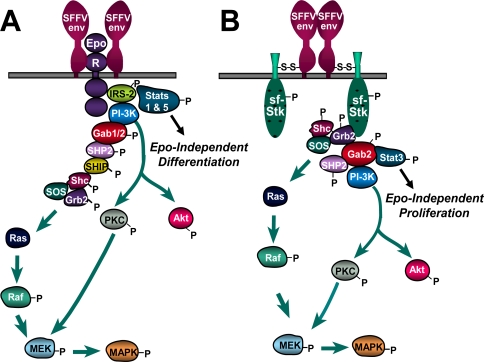
The envelope protein of SFFV interacts with the EpoR and sf-Stk to activate signal transduction pathways for growth, differentiation and survival of erythroid cells. **(A)** SFFV Env interacts with the EpoR, causing phosphorylation of the adaptor molecules IRS-2 and Gab1/2. This leads to activation of the Ras/Raf/MAPK and PI3-K pathways and phosphorylation of Stats 1 and 5, resulting in Epo-independent proliferation, differentiation and survival. **(B)** Covalent interaction of SFFV Env with sf-Stk results in activation of Grb2, Gab2 and Stat3. This leads to activation of the Ras/Raf/MAPK and PI-3K pathways and Epo-independent erythroid cell proliferation and survival. P = phosphorylation event; S-S = disulfide bond.

It is clear that the envelope protein of SFFV can interact not only with the EpoR but also with the tyrosine kinase sf-Stk, and these interactions cause constitutive activation of signal transduction pathways in SFFV-infected cells, as illustrated in Figure 5. In this figure, interaction of SFFV Env with the EpoR and sf-Stk are shown separately to illustrate the distinct signals generated, but it is likely that all three interact in erythroid cells, although the exact stoichiometry of the interactions is unknown. Most of the signals generated from the interaction of SFFV Env with either the EpoR or sf-Stk are common, and signals from both interactions may be required to raise the level of these common signals above a certain threshold to cause a biological effect. However, there are also some differences in the signals generated from the interaction of SFFV Env with either the EpoR or sf-Stk, and these may be responsible for the induction of Epo-independent proliferation *versus* differentiation by SFFV.

The interaction of SFFV Env with the EpoR appears to be required for the development of Epo-independent erythroid cell differentiation, not proliferation. Two signal transducing molecules activated by the interaction of SFFV Env with the EpoR, but not with sf-Stk, are Stat1 and Stat5 (Figure 5A). These transcription factors play a role in erythroid cell differentiation, and their activation in SFFV-P-infected, but not SFFV-A-infected, erythroid cells is likely to be responsible for the polycythemia uniquely induced by SFFV-P. The observation that SFFV-P can cause erythroblastosis but not polycythemia in Stat5 null mice [52] further supports a role for Stat5 in SFFV-P-mediated erythroid cell differentiation, but not proliferation. In contrast to the interaction of SFFV Env with the EpoR, its interaction with sf-Stk appears to be the major force driving the Epo-independent proliferation of erythroid cells. One of the signals that is activated by this interaction, and not interaction of SFFV Env with the EpoR, is the transcription factor Stat3 (Figure 5B). In contrast to Stats 1 and 5, Stat3 appears to be important for Epo-independent proliferation induced by SFFV. In addition, activation of Stat3, but not Stats 1 and 5, occurs in transformed fibroblasts co-expressing SFFV Env and sf-Stk, further indicating that Stat3 plays an important role downstream of sf-Stk in inducing cellular proliferation. Blocking Stat3 activity interferes with the ability of SFFV-P to induce splenomegaly in mice and Epo-independent erythroid colonies *in vitro*, demonstrating that Stat3 is required for the induction of Epo independence by SFFV [71,74]. Interestingly, Stat3 has been shown in a number of systems to be an oncogene, and it is constitutively activated in various human hematological malignancies [75]. One role for Stat3 during the first stage of SFFV-P-induced erythroleukemia may be to limit the differentiation of erythroid precursors through the induction of the myeloid transcription factor PU.1 [74], expanding the number of erythroid target cells for the virus. Erythroid hyperplasia induced by SFFV-A does not depend on Stat3-mediated PU.1 induction since this virus does not promote Epo-independent differentiation.

As mentioned previously, the biological effects of SFFV in mice are specific to the erythroid lineage, which are the only hematopoietic cells that express the EpoR. However, interaction of SFFV Env and the EpoR are not sufficient or essential for the development of Epo-independent erythroid hyperplasia by SFFV. Thus, the role that the EpoR plays in SFFV-induced erythroid hyperplasia is unclear. Perhaps its function is not to signal but to bring sf-Stk, by virtue of its interaction with SFFV Env, to a cellular compartment where it can interact with and activate signal transducing molecules. The erythroid specificity of SFFV-induced disease could also be the consequence of erythroid cells, being one of the few cells in mice that express sufficient levels of sf-Stk to be activated by SFFV Env. Lending support to this idea is the observation that a bicistronic vector co-expressing sf-Stk and SFFV Env causes non-erythroid diseases in mice, such as hemangiosarcomas and ovarian and uterine tumors [67]. Finally, SFFV may be activating other signal transduction pathways unique to erythroid cells that do not involve the EpoR. A recent study suggested that the rapid expansion of erythroid progenitors in the spleens of SFFV-infected mice required activation of the BMP-4-dependent stress erythropoiesis pathway [76]. This pathway does not appear to depend upon the EpoR but may involve sf-Stk.

## 4. Molecular Basis for the Transformation of Erythroid Cells by SFFV

**Figure 6:**
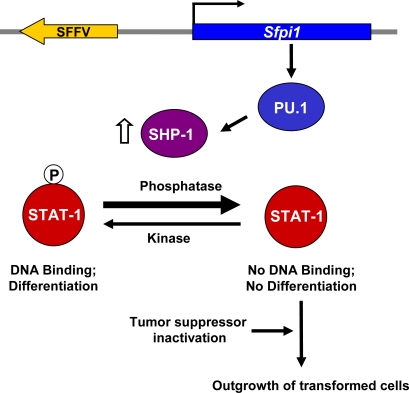
Transformation of erythroid cells by SFFV. SFFV integrates near the S*fpi1* gene, resulting in the expression of non-physiological levels of the transcription factor PU.1 in erythroid cells. PU.1 causes the upregulation of the tyrosine phosphatase SHP-1, which dephosphorylates Stat1. It is unknown whether PU.1 regulation of SHP-1 levels is direct or indirect. Phosphorylated Stat1, which is transported to the nucleus where it binds DNA, is thought to play a role in erythroid cell differentiation. Unphosphorylated Stat1 in SFFV-transformed cells cannot bind DNA, leading to a block in erythroid cell differentiation. Combined with the inactivation of the tumor suppressor gene *p53*, this leads to the outgrowth of transformed erythroid cells.

While interaction of the SFFV envelope protein with the EpoR and sf-Stk can account for the early polyclonal, Epo-independent proliferation of SFFV-infected erythroid cells, such interactions are not sufficient to induce a truly malignant disease. Additional molecular events are required. SFFV, like other retroviruses, is thought to transform erythroid cells by insertional mutagenesis. When malignant cells from SFFV-infected mice are examined for common sites of integration, almost all of them contain SFFV integrated at the S*fpi1* locus [77–79]. This results in non-physiological levels of the myeloid transcription factor PU.1 in erythroid cells, which is thought to abrogate the commitment of these cells to differentiate, leading to their transformation (Figure 6) [80].

Because SFFV does not block erythroid cell differentiation in the first stage of the disease, and some strains of the virus can even promote Epo-independent differentiation, it seemed likely that the genetic events associated with SFFV-induced transformation involved specifically blocking signals associated with erythroid cell differentiation. Stat transcription factors, particularly Stats 1 and 5, are thought to play a role in Epo-induced erythroid cell differentiation [52,81]. In the first stage of SFFV-P-induced disease, both Stats 1 and 5 are constitutively phosphorylated and bind DNA [52,82]. In contrast, Stat1 DNA binding is blocked in SFFV-transformed cells from the second stage of the disease, even in the presence of Epo [82]. Interferon-α is able to induce Stat1 activity in these SFFV-transformed cells, indicating that the block is specific to Stat activation by Epo or SFFV and not at the level of Stat1 expression. Also, the block is specific to activation of Stat1, not other signal transducing molecules activated in Stage 1, since both MAPK and Akt kinase are still constitutively activated in SFFV-transformed erythroid cells. There is a direct correlation in SFFV-infected erythroid cells between expression of PU.1 and inhibition of Stat1 DNA-binding activity [82], suggesting that PU.1 expression in erythroid cells may be causing the block in Stat1 activation.

Stat proteins are phosphorylated in the cytoplasm, form dimers and then are transported to the nucleus where they bind DNA. The block in Stat1 DNA binding in SFFV transformed cells is at the level of Stat1 tyrosine phosphorylation [82], implying a change in an enzyme that regulates phosphorylation status. Activation of Jak2, the major regulator of Stat tyrosine phosphorylation in erythroid cells, was not blocked in SFFV-transformed erythroid cells. Rather SFFV-transformed erythroid cells were shown to express high levels of the hematopoietic phosphatase SHP-1, which has previously been shown to negatively regulate the Jak-Stat pathway [83]. When SFFV-transformed cells were treated with the phosphatase inhibitor orthovanadate, both Epo-induced and constitutive Stat1 phosphorylation were restored, indicating that SHP-1 or another phosphatase is responsible for the block in Stat1 phosphorylation [82]. Interestingly, SHP-1 is downregulated in erythroid cells from PU.1 deficient mice [84], suggesting that PU.1 may directly or indirectly regulate SHP-1 expression. Furthermore, when SFFV-transformed cells are treated with chemicals to induce their differentiation, not only do the levels of PU.1 go down, but SHP-1 levels decrease and Stat1 DNA binding activity is restored [82].

While activation of PU.1 is an early and critical event in the transformation of erythroid cells by SFFV, another genetic event also occurs which is thought to enhance the outgrowth of SFFV-transformed cells: the loss of activity of the tumor suppressor p53 [85–88]. The loss of p53 activity is due either to SFFV integration, which blocks expression of the gene, or to spontaneous point mutations or deletions, resulting in an inactive protein. The outgrowth of SFFV-transformed erythroid cells occurs more rapidly in *p53* null mice or mice expressing a dominant-negative mutant *p53* transgene [86,89].

Thus, as illustrated in Figure 6, integration of the SFFV genome into the S*fpi1* locus in erythroid cells leads to transformation of erythroid cells by activating the myeloid transcription factor PU.1. Expression of inappropriately high levels of PU.1 in SFFV-infected erythroid cells results in an increase in the level of SHP-1, favoring dephosphorylation of Stat1 over its phosphorylation and blocking its DNA binding activity. Decreased levels of Stat1 DNA binding, cause a block in erythroid cell differentiation, favoring proliferation over differentiation. These events, combined with the inactivation of the *p53* tumor suppressor gene, are thought to favor the outgrowth of truly malignant, SFFV-infected erythroid cells.

## 5. Conclusions

**Figure 7:**
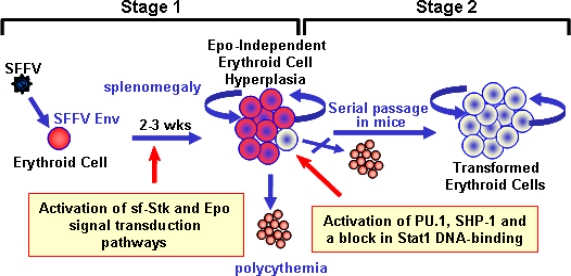
Molecular basis for the multi-stage erythroleukemia induced by SFFV. In Stage 1, expression of SFFV Env in erythroid cells results in the activation of sf-Stk and Epo signal transduction pathways, rapidly leading to a polyclonal expansion of Epo-independent erythroid cells and the development of polycythemia. Stage 2 occurs when an erythroid cell within this proliferating population expresses high levels of the transcription factor PU.1 due to SFFV integration, causing a block in erythroid cell differentiation. Serial passage of SFFV-infected splenic erythroblasts from Stage 1 through syngeneic mice allows the outgrowth of these rare SFFV-transformed cells, which are monoclonal and can be grown as erythroleukemia cell lines *in vitro.*

We can conclude, from studies to understand the molecular events occurring in SFFV-infected erythroid cells, that the virus causes a multi-stage erythroleukemia in mice by deregulating signal transduction pathways required for erythroid cell growth, differentiation, and survival (see Figure 7). The Epo-independent erythroid cell hyperplasia that occurs within weeks of virus infection is accomplished by interaction of the unique envelope glycoprotein of SFFV with the tyrosine kinase sf-Stk, leading to constitutive activation of various signal transduction pathways. SFFV Env can also interact with the EpoR, but this interaction drives Epo-independent differentiation, causing polycythemia, and does not appear to be essential for the development of erythroid hyperplasia. In fact, signals generated downstream from SFFV Env-activated sf-Stk can limit this differentiation, allowing for the rapid outgrowth of Epo-independent erythroid cells in the first stage of the disease. In the second stage, secondary genetic events caused by SFFV integration into host DNA cause a complete block in erythroid cell differentiation, even in the presence of Epo. This is the result of activation of the myeloid transcription factor PU.1, whose expression in erythroid cells interferes with signals required for erythroid cell differentiation, resulting in the outgrowth of fully transformed erythroid cells.

Recent studies have demonstrated an association of a recently identified human retrovirus with prostate cancer [90,91] and chronic fatigue syndrome [92]. This virus, xenotropic murine leukemia virus-related virus (XMRV), is closely related to SFFV and other murine leukemia viruses [91,93]. Although it is not known whether XMRV infection is the direct cause of these diseases, its similarity to SFFV and to other murine leukemia viruses that can cause malignant and neurological diseases in rodents suggests that it could use similar mechanisms to cause disease in humans. Because of its close relationship to the envelope protein of SFFV, it is possible that the XMRV envelope protein could also interact with a host tyrosine kinase to activate signal transduction pathways leading to cellular hyperplasia and transformation. Also, like SFFV, XMRV can integrate into the host genome [94], potentially activating oncogenes and leading to cellular transformation. Critical questions regarding the relationship of XMRV to human disease are being addressed in a number of laboratories; the knowledge of the molecular events that are responsible for the various stages of SFFV-induced erythroleukemia should be invaluable in designing *in vitro* and *in vivo* studies to determine the pathological potential of XMRV.

## Figures and Tables

**Table 1:** Signaling components of the Epo/EpoR pathway activated by SFFV. SFFV-P and SFFV-A, which have different effects on erythroid cell differentiation, are compared.

**Signaling Components of Epo/EpoR Pathway**	**SFFV-P**	**SFFV-A**	**Reference**
**Shc**	**+**	**+**	[47]
**Grb2**	**+**	**+**	[47]
**Ras**	**+**	**+**	[47]
**Raf-1**	**+**	**+**	[46]
**MEK**	**+**	**+**	[46]
**ERK 1/2**	**+**	**+**	[46]
**JNK**	**+**	**+**	[48]
**Stat1**	**+**	**−**	[50–52]
**Stat3**	**+**	**+**	[50–52]
**Stat5**	**+**	**−**	[50–52]
**PI 3-kinase**	**+**	**+**	[43]
**IRS-2**	**+**	**+**	[43]
**Gab1**	**+**	**+**	[43]
**Gab2**	**+**	**+**	[43]
**PKC**	**+**	**+**	[47]
**Akt**	**+**	**+**	[43]
**SHIP**	**+**	**+**	[43]

## References

[1] Friend C (1957). Cell-Free Transmission in Adult Swiss Mice of a Disease Having the Character of a Leukemia. J Exp Med.

[2] Aizawa S, Suda Y, Furuta Y, Yagi T, Takeda N, Watanabe N, Nagayoshi M, Ikawa Y (1990). Env-derived gp55 gene of Friend spleen focus-forming virus specifically induces neoplastic proliferation of erythroid progenitor cells. EMBO J.

[3] Wolff L, Ruscetti S (1988). The spleen focus-forming virus (SFFV) envelope gene, when introduced into mice in the absence of other SFFV genes, induces acute erythroleukemia. J Virol.

[4] Amanuma H, Katori A, Obata M, Sagata N, Ikawa Y (1983). Complete nucleotide sequence of the gene for the specific glycoprotein (gp55) of Friend spleen focus-forming virus. Proc Natl Acad Sci U S A.

[5] Clark SP, Mak TW (1983). Complete nucleotide sequence of an infectious clone of Friend spleen focus-forming provirus: gp55 is an envelope fusion glycoprotein. Proc Natl Acad Sci U S A.

[6] Wolff L, Scolnick E, Ruscetti S (1983). Envelope gene of the Friend spleen focus-forming virus: Deletion and insertions in 3′ gp70/p15E-encoding region have resulted in unique features in the primary structure of its protein product. Proc Natl Acad Sci U S A.

[7] Amanuma H, Watanabe N, Nishi M, Ikawa Y (1989). Requirement of the single base insertion at the 3′ end of the env-related gene of Friend spleen focus-forming virus for pathogenic activity and its effect on localization of the glycoprotein product (gp55). J Virol.

[8] Srinivas RV, Kilpatrick DR, Tucker S, Rui Z, Compans RW (1991). The hydrophobic membrane-spanning sequences of the gp52 glycoprotein are required for the pathogenicity of Friend spleen focus-forming virus. J Virol.

[9] Watanabe N, Nishi M, Ikawa Y, Amanuma H (1990). A deletion in the Friend spleen focus-forming virus env gene is necessary for its product (gp55) to be leukemogenic. J Virol.

[10] Watanabe N, Yugawa T, Ikawa Y, Amanuma H (1995). Both the changes of six amino acids and the C-terminal truncation caused by a one-base insertion in the defective env gene of Friend spleen focus-forming virus significantly affect the pathogenic activity of the encoded leukemogenic membrane glycoprotein (gp55). J Virol.

[11] Gliniak BC, Kabat D (1989). Leukemogenic membrane glycoprotein encoded by Friend spleen focus-forming virus: Transport to cell surfaces and shedding are controlled by disulfide-bonded dimerization and by cleavage of a hydrophobic membrane anchor. J Virol.

[12] Ruscetti SK, Linemeyer D, Feild J, Troxler D, Scolnick EM (1979). Characterization of a protein found in cells infected with the spleen focus-forming virus that shares immunological cross-reactivity with the gp70 found in mink cell focus-inducing virus particles. J Virol.

[13] Ruta M, Clarke S, Boswell B, Kabat D (1982). Heterogeneous metabolism and subcellular localization of a potentially leukemogenic membrane glycoprotein encoded by Friend erythroleukemia virus. Isolation of viral and cellular processing mutants. J Biol Chem.

[14] Srinivas RV, Compans RW (1983). Membrane association and defective transport of spleen focus-forming virus glycoproteins. J Biol Chem.

[15] Ferro FE, Kozak SL, Hoatlin ME, Kabat D (1993). Cell surface site for mitogenic interaction of erythropoietin receptors with the membrane glycoprotein encoded by Friend erythroleukemia virus. J Biol Chem.

[16] Li JP, Bestwick RK, Spiro C, Kabat D (1987). The membrane glycoprotein of Friend spleen focus-forming virus: Evidence that the cell surface component is required for pathogenesis and that it binds to a receptor. J Virol.

[17] Ruta M, Bestwick R, Machida C, Kabat D (1983). Loss of leukemogenicity caused by mutations in the membrane glycoprotein structural gene of Friend spleen focus-forming virus. Proc Natl Acad Sci U S A.

[18] Mager DL, Mak TW, Bernstein A (1981). Quantitative colony method for tumorigenic cells transformed by two distinct strains of Friend leukemia virus. Proc Natl Acad Sci U S A.

[19] Berger SA, Sanderson N, Bernstein A, Hankins WD (1985). Induction of the early stages of Friend erythroleukemia with helper-free Friend spleen focus-forming virus. Proc Natl Acad Sci U S A.

[20] Bestwick RK, Hankins WD, Kabat D (1985). Roles of helper and defective retroviral genomes in murine erythroleukemia: Studies of spleen focus-forming virus in the absence of helper. J Virol.

[21] Wolff L, Ruscetti S (1985). Malignant transformation of erythroid cells *in vivo* by introduction of a nonreplicating retrovirus vector. Science.

[22] Wolff L, Tambourin P, Ruscetti S (1986). Induction of the autonomous stage of transformation in erythroid cells infected with SFFV: Helper virus is not required. Virology.

[23] Horoszewicz JS, Leong SS, Carter WA (1975). Friend leukemia: Rapid development of erythropoietin-independent hematopoietic precursors. J Natl Cancer Inst.

[24] Liao SK, Axelrad AA (1975). Erythropoietin-independent erythroid colony formation *in vitro* by hemopoietic cells of mice infected with friend virus. Int J Cancer.

[25] Steinheider G, Seidel HJ, Kreja L (1979). Comparison of the biological effects of anemia inducing and polycythemia inducing Friend virus complex. Experientia.

[26] Tambourin PE, Wendling F, Jasmin C, Smadja-Joffe F (1979). The physiopathology of Friend leukemia. Leuk Res.

[27] Hoatlin ME, Kozak SL, Lilly F, Chakraborti A, Kozak CA, Kabat D (1990). Activation of erythropoietin receptors by Friend viral gp55 and by erythropoietin and down-modulation by the murine Fv-2r resistance gene. Proc Natl Acad Sci U S A.

[28] Li JP, D'Andrea AD, Lodish HF, Baltimore D (1990). Activation of cell growth by binding of Friend spleen focus-forming virus gp55 glycoprotein to the erythropoietin receptor. Nature.

[29] Ruscetti SK, Janesch NJ, Chakraborti A, Sawyer ST, Hankins WD (1990). Friend spleen focus-forming virus induces factor independence in an erythropoietin-dependent erythroleukemia cell line. J Virol.

[30] Constantinescu SN, Wu H, Liu X, Beyer W, Fallon A, Lodish HF (1998). The anemic Friend virus gp55 envelope protein induces erythroid differentiation in fetal liver colony-forming units-erythroid. Blood.

[31] Chung SW, Wolff L, Ruscetti SK (1989). Transmembrane domain of the envelope gene of a polycythemia-inducing retrovirus determines erythropoietin-independent growth. Proc Natl Acad Sci U S A.

[32] Wolff L, Kaminchik J, Hankins WD, Ruscetti SK (1985). Sequence comparisons of the anemia- and polycythemia-inducing strains of Friend spleen focus-forming virus. J Virol.

[33] Constantinescu SN, Liu X, Beyer W, Fallon A, Shekar S, Henis YI, Smith SO, Lodish HF (1999). Activation of the erythropoietin receptor by the gp55-P viral envelope protein is determined by a single amino acid in its transmembrane domain. EMBO J.

[34] Fang C, Choi E, Nie L, Li JP (1998). Role of the transmembrane sequence of spleen focus-forming virus gp55 in erythroleukemogenesis. Virology.

[35] Rosenberg N, Jolicoeur P, Coffin JM, Hughes SH, Varmus HE (1997). Retroviral pathogenesis. Retroviruses.

[36] Lilly F (1970). Fv-2: Identification and location of a second gene governing the spleen focus response to Friend leukemia virus in mice. J Natl Cancer Inst.

[37] Persons DA, Paulson RF, Loyd MR, Herley MT, Bodner SM, Bernstein A, Correll PH, Ney PA (1999). Fv2 encodes a truncated form of the Stk receptor tyrosine kinase. Nat Genet.

[38] Friend C, Haddad JR (1960). Tumor formation with transplants of spleen or liver from mice with virus-induced leukemia. J Natl Cancer Inst.

[39] Friend C, Pateleia MC, DeHarven E (1966). Erythrocytic maturation *in vitro* of murine (Friend) virus-induced leukemia cells. Natl Cancer Inst Monogr.

[40] Marks PA, Rifkind RA (1978). Erythroleukemic differentiation. Annu Rev Biochem.

[41] Friend C, Sher W, Holland JG, Sato T (1971). Hemoglobin synthesis in murine virus-induced leukemai cells *in vitro*: Stimulation of erythroid differentiation by dimethylsulfoxide. Proc Natl Acad Sci U S A.

[42] Richmond TD, Chohan M, Barber DL (2005). Turning cells red: Signal transduction mediated by erythropoietin. Trends Cell Biol.

[43] Nishigaki K, Hanson C, Ohashi T, Thompson D, Muszynski K, Ruscetti S (2000). Erythroid cells rendered erythropoietin independent by infection with Friend spleen focus-forming virus show constitutive activation of phosphatidylinositol 3-kinase and Akt kinase: Involvement of insulin receptor substrate-related adapter proteins. J Virol.

[44] Verdier F, Chretien S, Billat C, Gisselbrecht S, Lacombe C, Mayeux P (1997). Erythropoietin induces the tyrosine phosphorylation of insulin receptor substrate-2. An alternate pathway for erythropoietin-induced phosphatidylinositol 3-kinase activation. J Biol Chem.

[45] Zang H, Sato K, Nakajima H, McKay C, Ney PA, Ihle JN (2001). The distal region and receptor tyrosines of the Epo receptor are non-essential for *in vivo* erythropoiesis. EMBO J.

[46] Muszynski KW, Ohashi T, Hanson C, Ruscetti SK (1998). Both the polycythemia- and anemia-inducing strains of Friend spleen focus-forming virus induce constitutive activation of the Raf-1/mitogen-activated protein kinase signal transduction pathway. J Virol.

[47] Muszynski KW, Thompson D, Hanson C, Lyons R, Spadaccini A, Ruscetti SK (2000). Growth factor-independent proliferation of erythroid cells infected with Friend spleen focus-forming virus is protein kinase C dependent but does not require Ras-GTP. J Virol.

[48] Nishigaki K, Hanson C, Thompson D, Yugawa T, Ruscetti S (2005). Activation of the Jun N-terminal kinase pathway by Friend spleen focus-forming virus and its role in the growth and survival of Friend virus-induced erythroleukemia cells. J Virol.

[49] Finkelstein LD, Ney PA, Liu QP, Paulson RF, Correll PH (2002). Sf-Stk kinase activity and the Grb2 binding site are required for Epo-independent growth of primary erythroblasts infected with Friend virus. Oncogene.

[50] Ohashi T, Masuda M, Ruscetti SK (1995). Induction of sequence-specific DNA-binding factors by erythropoietin and the spleen focus-forming virus. Blood.

[51] Ohashi T, Masuda M, Ruscetti SK (1997). Constitutive activation of Stat-related DNA-binding proteins in erythroid cells by the Friend spleen focus-forming virus. Leukemia.

[52] Zhang J, Randall MS, Loyd MR, Li W, Schweers RL, Persons DA, Rehg JE, Noguchi CT, Ihle JN, Ney PA (2006). Role of erythropoietin receptor signaling in Friend virus-induced erythroblastosis and polycythemia. Blood.

[53] Ruscetti S, Hanson C (2004). National Cancer Institute, Frederick, MD, USA.

[54] Socolovsky M, Fallon AE, Wang S, Brugnara C, Lodish HF (1999). Fetal anemia and apoptosis of red cell progenitors in Stat5a^−/−^5b^−/−^ mice: A direct role for Stat5 in Bcl-X_L_ induction. Cell.

[55] Ruscetti S, Yugawa T (2005). National Cancer Institute, Frederick, MD, USA.

[56] Umehara D, Watanabe S, Ochi H, Anai Y, Ahmed N, Kannagi M, Hanson C, Ruscetti S, Nishigaki K (2010). Role of phosphatidylinositol 3-kinase in friend spleen focus-forming virus-induced erythroid disease. J Virol.

[57] Tsushima H, Urata Y, Miyazaki Y, Fuchigami K, Kuriyama K, Kondo T, Tomonaga M (1997). Human erythropoietin receptor increases GATA-2 and Bcl-x_L_ by a protein kinase C-dependent pathway in human erythropoietin-dependent cell line AS-E2. Cell Growth Differ.

[58] Datta SR, Dudek H, Tao X, Masters S, Fu H, Gotoh Y, Greenberg ME (1997). Akt phosphorylation of BAD couples survival signals to the cell-intrinsic death machinery. Cell.

[59] Casadevall N, Lacombe C, Muller O, Gisselbrecht S, Mayeux P (1991). Multimeric structure of the membrane erythropoietin receptor of murine erythroleukemia cells (Friend cells). Cross-linking of erythropoietin with the spleen focus-forming virus envelope protein. J Biol Chem.

[60] Zon LI, Moreau JF, Koo JW, Mathey-Prevot B, D'Andrea AD (1992). The erythropoietin receptor transmembrane region is necessary for activation by the Friend spleen focus-forming virus gp55 glycoprotein. Mol Cell Biol.

[61] Constantinescu SN, Keren T, Russ WP, Ubarretxena-Belandia I, Malka Y, Kubatzky KF, Engelman DM, Lodish HF, Henis YI (2003). The erythropoietin receptor transmembrane domain mediates complex formation with viral anemic and polycythemic gp55 proteins. J Biol Chem.

[62] Witthuhn BA, Quelle FW, Silvennoinen O, Yi T, Tang B, Miura O, Ihle JN (1993). JAK2 associates with the erythropoietin receptor and is tyrosine phosphorylated and activated following stimulation with erythropoietin. Cell.

[63] Subramanian A, Hegde S, Correll PH, Paulson RF (2006). Mutation of the Lyn tyrosine kinase delays the progression of Friend virus induced erythroleukemia without affecting susceptibility. Leuk Res.

[64] Wang MH, Iwama A, Skeel A, Suda T, Leonard EJ (1995). The murine stk gene product, a transmembrane protein tyrosine kinase, is a receptor for macrophage-stimulating protein. Proc Natl Acad Sci U S A.

[65] Waltz SE, Toms CL, McDowell SA, Clay LA, Muraoka RS, Air EL, Sun WY, Thomas MB, Degen SJ (1998). Characterization of the mouse Ron/Stk receptor tyrosine kinase gene. Oncogene.

[66] Iwama A, Okano K, Sudo T, Matsuda Y, Suda T (1994). Molecular cloning of a novel receptor tyrosine kinase gene, STK, derived from enriched hematopoietic stem cells. Blood.

[67] Rulli K, Yugawa T, Hanson C, Thompson D, Ruscetti S, Nishigaki K (2004). *Ex vivo* and *in vivo* biological effects of a truncated form of the receptor tyrosine kinase Stk when activated by interaction with the friend spleen focus-forming virus envelope glycoprotein or by point mutation. J Virol.

[68] Nishigaki K, Thompson D, Hanson C, Yugawa T, Ruscetti S (2001). The envelope glycoprotein of friend spleen focus-forming virus covalently interacts with and constitutively activates a truncated form of the receptor tyrosine kinase Stk. J Virol.

[69] He S, Ni S, Hegde S, Wang X, Sharda DR, August A, Paulson RF, Hankey PA (2010). Activation of the N-terminally truncated form of the Stk receptor tyrosine kinase Sf-Stk by Friend virus-encoded gp55 is mediated by cysteine residues in the ecotropic domain of gp55 and the extracellular domain of Sf-Stk. J Virol.

[70] Teal HE, Ni S, Xu J, Finkelstein LD, Cheng AM, Paulson RF, Feng GS, Correll PH (2006). GRB2-mediated recruitment of GAB2, but not GAB1, to SF-STK supports the expansion of Friend virus-infected erythroid progenitor cells. Oncogene.

[71] Ni S, Zhao C, Feng GS, Paulson RF, Correll PH (2007). A novel Stat3 binding motif in Gab2 mediates transformation of primary hematopoietic cells by the Stk/Ron receptor tyrosine kinase in response to Friend virus infection. Mol Cell Biol.

[72] Nishigaki K, Hanson C, Jelacic T, Thompson D, Ruscetti S (2005). Friend spleen focus-forming virus transforms rodent fibroblasts in cooperation with a short form of the receptor tyrosine kinase Stk. Proc Natl Acad Sci U S A.

[73] Jelacic TM, Thompson D, Hanson C, Cmarik J, Nishigaki K, Ruscetti S (2008). The tyrosine kinase sf-stk and its downstream signals are required for maintenance of friend spleen focus-forming virus-induced fibroblast transformation. J Virol.

[74] Hegde S, Ni S, He S, Yoon D, Feng GS, Watowich SS, Paulson RF, Hankey PA (2009). Stat3 promotes the development of erythroleukemia by inducing Pu.1 expression and inhibiting erythroid differentiation. Oncogene.

[75] Turkson J, Jove R (2000). STAT proteins: Novel molecular targets for cancer drug discovery. Oncogene.

[76] Subramanian A, Hegde S, Porayette P, Yon M, Hankey P, Paulson RF (2008). Friend virus utilizes the BMP4-dependent stress erythropoiesis pathway to induce erythroleukemia. J Virol.

[77] Moreau-Gachelin F, Tavitian A, Tambourin P (1988). Spi-1 is a putative oncogene in virally induced murine erythroleukaemias. Nature.

[78] Paul R, Schuetze S, Kozak SL, Kabat D (1989). A common site for immortalizing proviral integrations in Friend erythroleukemia: Molecular cloning and characterization. J Virol.

[79] Paul R, Schuetze S, Kozak SL, Kozak CA, Kabat D (1991). The Sfpi-1 proviral integration site of Friend erythroleukemia encodes the ets-related transcription factor Pu.1. J Virol.

[80] Schuetze S, Paul R, Gliniak BC, Kabat D (1992). Role of the PU.1 transcription factor in controlling differentiation of Friend erythroleukemia cells. Mol Cell Biol.

[81] Halupa A, Bailey ML, Huang K, Iscove NN, Levy DE, Barber DL (2005). A novel role for STAT1 in regulating murine erythropoiesis: Deletion of STAT1 results in overall reduction of erythroid progenitors and alters their distribution. Blood.

[82] Nishigaki K, Hanson C, Ohashi T, Spadaccini A, Ruscetti S (2006). Erythroblast transformation by the friend spleen focus-forming virus is associated with a block in erythropoietin-induced STAT1 phosphorylation and DNA binding and correlates with high expression of the hematopoietic phosphatase SHP-1. J Virol.

[83] Klingmuller U, Lorenz U, Cantley LC, Neel BG, Lodish HF (1995). Specific recruitment of SH-PTP1 to the erythropoietin receptor causes inactivation of JAK2 and termination of proliferative signals. Cell.

[84] Fisher RC, Slayton WB, Chien C, Guthrie SM, Bray C, Scott EW (2004). PU.1 supports proliferation of immature erythroid progenitors. Leuk Res.

[85] Ben David Y, Prideaux VR, Chow V, Benchimol S, Bernstein A (1988). Inactivation of the p53 oncogene by internal deletion or retroviral integration in erythroleukemic cell lines induced by Friend leukemia virus. Oncogene.

[86] Lavigueur A, Bernstein A (1991). p53 transgenic mice: Accelerated erythroleukemia induction by Friend virus. Oncogene.

[87] Mowat M, Cheng A, Kimura N, Bernstein A, Benchimol S (1985). Rearrangements of the cellular p53 gene in erythroleukaemic cells transformed by Friend virus. Nature.

[88] Munroe DG, Peacock JW, Benchimol S (1990). Inactivation of the cellular p53 gene is a common feature of Friend virus-induced erythroleukemia: Relationship of inactivation to dominant transforming alleles. Mol Cell Biol.

[89] Prasher JM, Elenitoba-Johnson KS, Kelley LL (2001). Loss of p53 tumor suppressor function is required for *in vivo* progression of Friend erythroleukemia. Oncogene.

[90] Schlaberg R, Choe DJ, Brown KR, Thaker HM, Singh IR (2009). XMRV is present in malignant prostatic epithelium and is associated with prostate cancer, especially high-grade tumors. Proc Natl Acad Sci U S A.

[91] Urisman A, Molinaro RJ, Fischer N, Plummer SJ, Casey G, Klein EA, Malathi K, Magi-Galluzzi C, Tubbs RR, Ganem D, Silverman RH, DeRisi JL (2006). Identification of a novel Gammaretrovirus in prostate tumors of patients homozygous for R462Q RNASEL variant. PLoS Pathog.

[92] Lombardi VC, Ruscetti FW, Das Gupta J, Pfost MA, Hagen KS, Peterson DL, Ruscetti SK, Bagni RK, Petrow-Sadowski C, Gold B, Dean M, Silverman RH, Mikovits JA (2009). Detection of an infectious retrovirus, XMRV, in blood cells of patients with chronic fatigue syndrome. Science.

[93] Lee K, Jones KS (2010). The path well traveled: Using mammalian retroviruses to guide research on XMRV. Mol Interv.

[94] Kim S, Kim N, Dong B, Boren D, Lee SA, Das Gupta J, Gaughan C, Klein EA, Lee C, Silverman RH, Chow SA (2008). Integration site preference of xenotropic murine leukemia virus-related virus, a new human retrovirus associated with prostate cancer. J Virol.

